# Institutional delivery service utilization and associated factors among mothers who gave birth in the last 12 months in Sekela District, North West of Ethiopia: *A community - based cross sectional study*

**DOI:** 10.1186/1471-2393-12-74

**Published:** 2012-07-31

**Authors:** Alemayehu Shimeka Teferra, Fekadu Mazengia Alemu, Solomon Meseret Woldeyohannes

**Affiliations:** 1 Department of Nursing, Health Sciences College, Madawalabu University, Bale Goba, Ethiopia; 2 Department of Midwifery, College of Medicine and Health Sciences, University of Gondar, Gondar, Ethiopia; 3 Department of Epidemiology and Biostatistics, College of Medicine and Health Sciences, University of Gondar, Gondar, Ethiopia

**Keywords:** Institutional delivery service utilization, Preferred place of delivery, Sekela District

## Abstract

**Background:**

Reduction of maternal mortality is a global priority particularly in developing countries including Ethiopia where maternal mortality ratio is one of the highest in the world. The key to reducing maternal mortality ratio and improving maternal health is increasing attendance by skilled health personnel throughout pregnancy and delivery. However, delivery service is significantly lower in Amhara Regional State, Ethiopia. Therefore, this study aimed to assess factors affecting institutional delivery service utilization among mothers who gave birth in the last 12 months in Sekela District, Amhara Region, Ethiopia.

**Methods:**

Community-based cross-sectional study was conducted among mothers with birth in the last 12 months during August, 2010. Multistage sampling technique was used to select 371 participants. A pre tested and structured questionnaire was used to collect data. Bivariate and multivariate data analysis was performed using SPSS version 16.0 software.

**Results:**

The study indicated that 12.1% of the mothers delivered in health facilities. Of 87.9% mothers who gave birth at home, 80.0% of them were assisted by family members and relatives. The common reasons for home delivery were closer attention from family members and relatives (60.9%), home delivery is usual practice (57.7%), unexpected labour (33.4%), not being sick or no problem at the time of delivery (21.6%) and family influence (14.4%). Being urban resident (AOR [95% CI] = 4.6 [1.91, 10.9]), ANC visit during last pregnancy (AOR [95% CI] = 4.26 [1.1, 16.4]), maternal education level (AOR [95%CI] =11.98 [3.36, 41.4]) and knowledge of mothers on pregnancy and delivery services (AOR [95% CI] = 2.97[1.1, 8.6]) had significant associations with institutional delivery service utilization.

**Conclusions:**

Very low institutional delivery service utilization was observed in the study area. Majority of the births at home were assisted by family members and relatives. ANC visit and lack of knowledge on pregnancy and delivery services were found to be associated with delivery service utilization. Strategies with focus on increasing ANC uptake and building knowledge of the mothers and their partners would help to increase utilization of the service. Training and assigning skilled attendants at Health Post^a^ level to provide skilled home delivery would improve utilization of the service.

## Background

Maternal mortality remains a major challenge to health systems worldwide. According to assessment of trends in maternal mortality for 181 countries from 1980–2008, it was estimated to be 342,900 maternal deaths worldwide in 2008 decreasing from 526,300 in 1980. More than 50% of all maternal deaths were only from six countries in 2008 (India, Nigeria, Pakistan, Afghanistan, Ethiopia, and the Democratic Republic of Congo) [1]. Maternal deaths have both direct and indirect causes. About 80% of maternal deaths are due to causes directly related to pregnancy and childbirth [2]. Worldwide, the major causes of maternal mortality are haemorrhage (24%), infection (15%), unsafe abortion (13%), prolonged labour (12%) and eclampsia (12%) whereas primary causes of maternal mortality in Africa are haemorrhage (34%), other direct causes (17%), infection (10%), hypertensive disorders (9%) and obstructed labour (4%), abortion (4%) and anaemia (4%) [1].

Major causes of maternal deaths in Ethiopia are similar to most developing countries such as infection, haemorrhage, obstructed labour, abortion and hypertension in pregnancy [3]*.* At the health facility level haemorrhage (PPH) is responsible for 11% of all maternal deaths due to direct obstetric complications. The major direct obstetric complications include haemorrhage (APH & PPH), prolonged/obstructed labour and ruptured uterus, severe pre-eclampsia and eclampsia, sepsis, complications of abortion and ectopic pregnancy which account for 69% of the deaths. The proportion of deaths due to PPH that occurred in facilities is most likely due to the fact that over 90% of births take place at home, and women with PPH may not be arriving at a health facility in time [4].

One of the objectives of the United Nations Millennium Development Goals (MDGs) was to reduce MMR by an average of 5.5% every year over the period 1990–2015. At the global level, MMR decreased by less than 1% per year between 1990 and 2005 far below 5.5% to reach the target of MGD [5]*.* Of all 8 MDGs, countries have made the least progress toward MDG 5 [6]. Most Sub- Saharan African countries are not on track for meeting the targets pertaining to MMR. Recent estimates suggest that the average annual rate of reduction in MMR in SSA countries is less than 1% [7]. As Ethiopian EDHS 2011 has shown, the MMR was 676 per 100,000 live births for the seven year period preceding the survey which is not significantly different from EDHS 2005 report (673 per 100,000 live births) [8].

The proportion of women who delivered with the assistance of a skilled birth attendant is one of the indicators in meeting the fifth MDG. In almost all countries where health professionals attend more than 80% of deliveries, MMR is below 200 per 100,000 live births [9]. However, birth with skilled attendance was low in Southern Asia (40%) and SSA (47%), the two regions with the greatest number of maternal deaths [10].

In Ethiopia, the proportions of births attended by skilled personnel are very much lower than SSA. Even for women who have access to the services, the proportion of births occurring in health facilities is very low. Only 6% of births were delivered in health facilities and, there is no significant difference in proportions of delivery service utilization between EDHS 2000 and 2005; however this figure moderately increased to 10% in EDHS 2011. Twenty eight percent of mothers delivered by TBAs; while the majority of births were attended by a relative or some other person (61%) and 5% of all births were delivered without any type of assistance at all [8,11].

The National Baseline Assessment for Emergency Obstetric and Newborn Care in 2008 indicated that skilled delivery service utilization was 7%. The majority of Ethiopian women give birth at home without skilled attendants [4]. According to the Ethiopian health system policy, the health service delivery structure has a four-tier system. This includes Primary Health Care Unit (PHCU), District Hospital, Zonal Hospital and Specialized Hospital. The PHCU includes one health center and five health posts. Each health post provides services to 5,000 people and is staffed by health extension workers. One health center serves a total 25,000 people and is led by a health officer. The PHCU provides comprehensive, integrated and community-based preventive and basic curative services. District Hospital functions as a referral and training center for ten PHCUs. Zonal Hospitals provide specialist services and training while Specialized Hospitals provide comprehensive specialist services, and in some instances serve as centers for research and post basic training. Maternal health services, especially delivery care, are given in health centers and at hospital level but not in health posts [12].

Institutional delivery service utilization in Amhara region was about 3.5% in EDHS 2005 which in turn was much lower than the national level [11]. In EDHS 2011 it has increased to 10.2% [8]. Hence, this study was conducted to determine the status of skilled delivery service utilization and associated factors in Sekela District, Amhara Regional State, Ethiopia.

## Methods

### Study design and period

Community-based cross-sectional study was conducted during the month of August, 2010 in Sekela District. Sekela District is one of the 138 districts of Amhara Region. It is found in West Gojjam Administrative Zone. The capital of Sekela is Gish Abay. It is located 175 km and 460 km far from Bahir Dar and Addis Ababa which are capital cities of the Amhara Region and Ethiopia, respectively. According to CSA 2007 census result, the district has a total population of 138,652; with sex ratio of 1.01:1. The number of child bearing women (15–49 years) is 28,544 [13]. The prevalence of any method of family planning methods in the district is 37%. About 90% of the population of the district are rural dwellers. In the district, there were five currently functional and three developing Health Centers. There were 119 health workers in the District with only two midwives in the district. In addition, 27 health posts were found with a total of 61 Health Extension Workers (HEWs). Since HEWs were not trained on delivery services, they were not allowed to assist delivery based on the current policy of the country [14].

### Sample size and sampling techniques

Childbearing women who gave birth in the last 12 months in the District, regardless of their birth outcome, were included in the sample. The required sample size of eligible mothers for the study was determined using the formula to estimate single population proportion. The following assumptions were made while calculating the sample size. A 95% probability of obtaining the population proportion of mothers who gave birth at the health institutions within 4% margin of error and population proportion of mothers who gave birth at the health institutions assumed to be 7% taken from National Baseline Assessment for Emergency Obstetric and Newborn Care Ethiopia in 2008 [4]. Using the Statistical Package Epi_Info version 2000 and considering a design effect of 2 owing to the use of multistage cluster sampling [15,16], the required size was 314. Expecting a 15% non response rate, the final sample size was calculated to be 371. Two stage cluster sampling technique was used to select the study units. Initially, the study area was divided into two clusters, urban and rural Kebeles^b^. There were two urban and 27 rural Kebeles during the study time. One urban Kebele and four rural Kebeles were selected using lottery method. On the selected five Kebeles house-to-house visit was carried out to identify households with eligible women and 819 households were found fulfilling the eligibility criteria. Households from each kebele were selected again by systematic random sampling with a sampling interval of 2 using list prepared during the house to house visit. If the houses were closed or the mothers were not present at the time of data collection, frequent visits were made until we could communicate them throughout the data collection. The next houses were considered in place of the houses which could not be accessed for collecting mothers’ data regarding institutional delivery service utilization. If there were more than one mother within the same household lottery method was used to select the one to be included in the sample.

### Data collection procedures

Data were collected on mothers’ age, marital status, place of residence, family income, educational status, occupation, educational status of the husband, occupation of the husband, age difference between the mother and the husband, institutional delivery service utilization, distance from health facility, family size, communication media possession and obstetrics variables such as age at first pregnancy, ANC visit, parity, gravidity, history of abortion and still birth, knowledge and attitude of the mother towards the ANC and delivery services in health facilities.

Structured and pre-tested questionnaire was prepared first in English and then translated into Amharic, the local language. Six Health Science College Students had conducted the face to face interviews and two nurses had supervised the data collection process. Training was given to the data collectors and supervisors before the actual data collection regarding the aim of study, and data collection tool and procedures going through the questionnaires question by question. In addition, the training also focused on the art of interviewing and clarifying questions that were unclear to the respondents. Data collectors were peer interviewed.

### Data processing and analysis

Collected questionnaires were checked visually for completeness, coded and entered into SPSS version 16.0 software package. Frequency run and double data entry on 10% of the questionnaires were performed to check data entry errors. Binary and multiple logistic regressions were run to assess the putative associations of various factors with institutional delivery service utilization. The results were presented in the form of tables, figures and summary statistics. The strength of association of predictor variables with institutional delivery service utilization was assessed using odds ratio with 95% confidence interval.

Ethical clearance was obtained from Institutional Review Board (IRB) of University of Gondar. Permission to conduct the study was also obtained from the Regional Health Bearuo and Sekela District Health Office. Informed consent was obtained from each study participant. Each respondent was informed about the purpose of the study that the findings of the study will inform policy makers and other concerned bodies. Any involvement in the study was after their complete verbal consent was obtained. Mothers were also informed that all data obtained from them would be kept confidential by using codes instead of any personal identifiers.

## Results

### Respondents’ Socio-demographics

A total of 371 mothers who gave birth in the last 12 months were interviewed; of these, 296 (79.8%) were rural and 75 (20.2%) were urban residents with 100% participation rate.

The mean age of the respondents was 27.41 ± 5.88 SD. Two hundred twenty seven (61.3%) of the mothers were in the age range of 20–29 years. Regarding their marital status, majority 338 (91.1%) and 17 (4.6%) of them were married and separated, respectively. Only 41 (8.9%) of the mothers attended primary and secondary education while 304 (81.9%) of the mothers were unable to read and write.

Among the respondents, 296 (79.8%) of mothers were housewives. The rest 20.2% included employed mothers, tea and tella *(local beer with low alcohol content)* sellers, daily labourers, students and others in occupational status. More than half their husbands (52.7%) were in age group of 30–39 years of age, 24.2% were between 40–49 years, 17.7% were between 20–29 years and the rest 5.4% were 49 years and above. One hundred seventy (47.9%) of the husbands were able to read and write, and 146 (41.1%) of them were unable to read and write. As to the husband’s occupational status, the majority 263 (74.1%) were farmers.

Economically, 97 (26.1%) of the households had monthly income of between 60–408 ETB and 93 (25.1%) had 694–987 ETB monthly income based on quartile classification. One hundred four (28.0%) of the respondents had radio and 27 (7.3%) had TV, and the other 64.7% did not possess media of communication. Concerning the time they travelled on foot to reach the nearby health center, 268 (72.2%) of them said less than one hour, 72 (19.4%) said between one to two hours and 31 (8.4%) said more than two hours. Two hundred fourteen (57.7%) of mothers had family size of 2–5 and 157(42.3%) had more than five individuals within the household [Table 1].

**Table 1:** Socio-demographic characteristics of mothers (N = 371) in Sekela district, North West Ethiopia, August, 2010

**Socio demographic Variables**	**N (%)**
**Place of residence**	
Urban	75(20.2)
Rural	296(79.8)
**Age of mother at interview** (Mean, SD,27.41 ± 5.88)	
15-19	19(5.1)
20-24	91(24.6)
25-29	136(36.7)
30-34	65(17.5)
35-39	45(12.1)
40+	15(4.0)
**Marital Status of mother**	
Married	338(91.1)
Divorced/widowed/single	16(4.3)
Separated	17(4.6)
**Educational Status of mother**	
Unable to read & write	304(81.8)
Able to read & write	34(9.2)
Primary education(1–8)	10(2.7)
Secondary education and above (9-12+)	23(6.2)
**Educational Status of husband**(n = 355)	
Unable to read & write	146(41.1)
Able to read & write	170(47.9)
Primary education(1–8)	16(4.5)
Secondary education and above (9-12+)	23(6.5)
**Occupational status of mother**	
House wife	296(79.8)
Gov’t employed	14(3.8)
Tea and tella† seller	28(7.5)
Merchant	6(1.6)
Daily labourers	15(4.0)
Students	8(2.2)
Others*	4(1.1)
**Occupational status of husband**(n = 355)	
Farmer	263(74.1)
Employed (Government & Private)	21(5.9)
Merchant	33(9.3)
Daily labourers	23(6.5)
Others**	15(4.2)
**Income in month in ETB**	
60-408	97(26.1)
409-693	90(24.3)
694-987	93(25.1)
> = 988	91(24.5)
**Time taken to nearby health center**	
<1 hour	268(72.2)
1-2 hours	72(19.4)
>2 hours	31(8.4)
**Family size**	
2-5	214(57.7)
>5	157(42.5)

*Rented House workers ** Carpenter, Construction worker † *Local low alcohol drink Construction worker* † *Local low alcohol drink* .

### Obstetric characteristics of the respondents

Two hundred ninety seven (80.1%) of the mothers became pregnant before the age of 20 years. The minimum and maximum ages at first pregnancy were 15 and 27 years with mean 18.2 years and ± 2.71 SD. One hundred eighty nine (50.9%) of the mothers were gravida two to four and 113 (30.5%) of them were gravida five and above, more than half of the respondents (52.8%) were between para two and four while 28.6% were parity five and above.

Before the last pregnancy, 167 (44.0%) of the respondents had ever visited health facilities during pregnancy and 248 (66.8%) of them visited health facilities for ANC purposes during their last pregnancy. Among the mothers who attended ANC, 165 (66.5%) of them visited health facilities two to three times [Table 2].

**Table 2:** Obstetric characteristics of respondents (N = 371) in Sekela district, North West Ethiopia, August, 2010

**Variables**	**N (%)**
**Age at first union**	
<15	24(6.5)
15-19	278(74.9)
20+	69(18.6)
Age at first pregnancy	
<20	297(80.1)
> = 20	74(19.9)
**Gravidity**	
1	69(18.6)
2-4	189(50.9)
> = 5	113(30.5)
Parity	
1	69(18.6)
2-4	196(52.8)
> = 5	106(28.6)
**Abortion in life time**	
Yes	34(9.2)
No	340(90.8)
**Still birth in life time**	
Yes	29(7.8)
No	342(92.2)
**ANC visit during previous pregnancy**	
Yes	167(44.0)
No	204(56.0)
**ANC visit during last pregnancy**	
Yes	248(66.8)
No	123(33.2)
**Number ANC visit during last pregnancy**	
Only one	42(16.9)
Two to three	165(66.5)
Four and above	4116.5()
**Place of last 12 month delivery**	
Health facility	45(12.1)
Home	326(87.9)
Assistant during last Delivery at home **(n = 326)**	
Family member	274(80.0)
My mother	13(3.98)
TBA (Untrained)	9(2.8)
Health worker	2(0.6)
No one/Myself	20(6.1)
Others*	8(2.5)

* *Neighbors, Non relatives.*

### Institutional delivery service utilization

**Figure 1:**
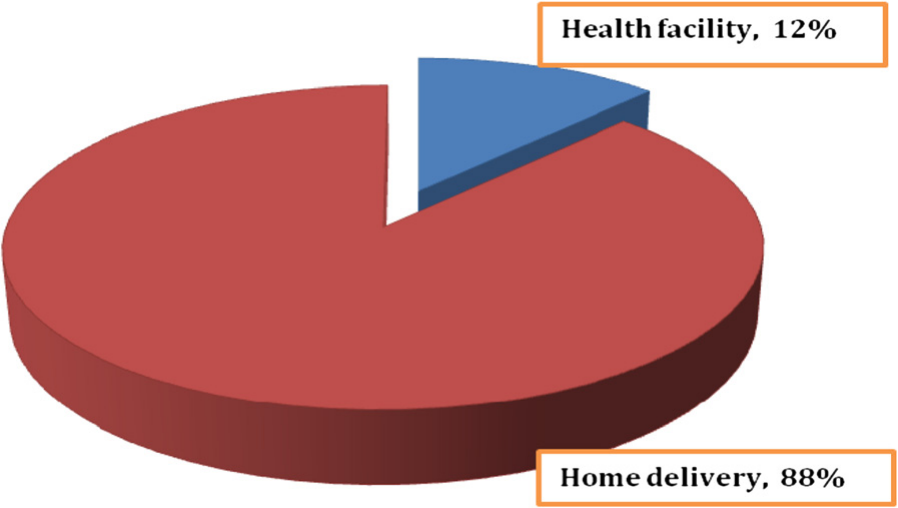
The proportion of births attended health facilities and home among mothers, Sekela district, North West Ethiopia, Aug. 2010.

Of the total respondents, only 45 (12.1%) of them gave birth at health facilities and majority of them (87.9%) delivered at home claiming that home was best place for giving birth [Figure 1]. Out of those mothers who delivered at home, 274 (80.0%) were assisted by family members [Table 2].

**Figure 2:**
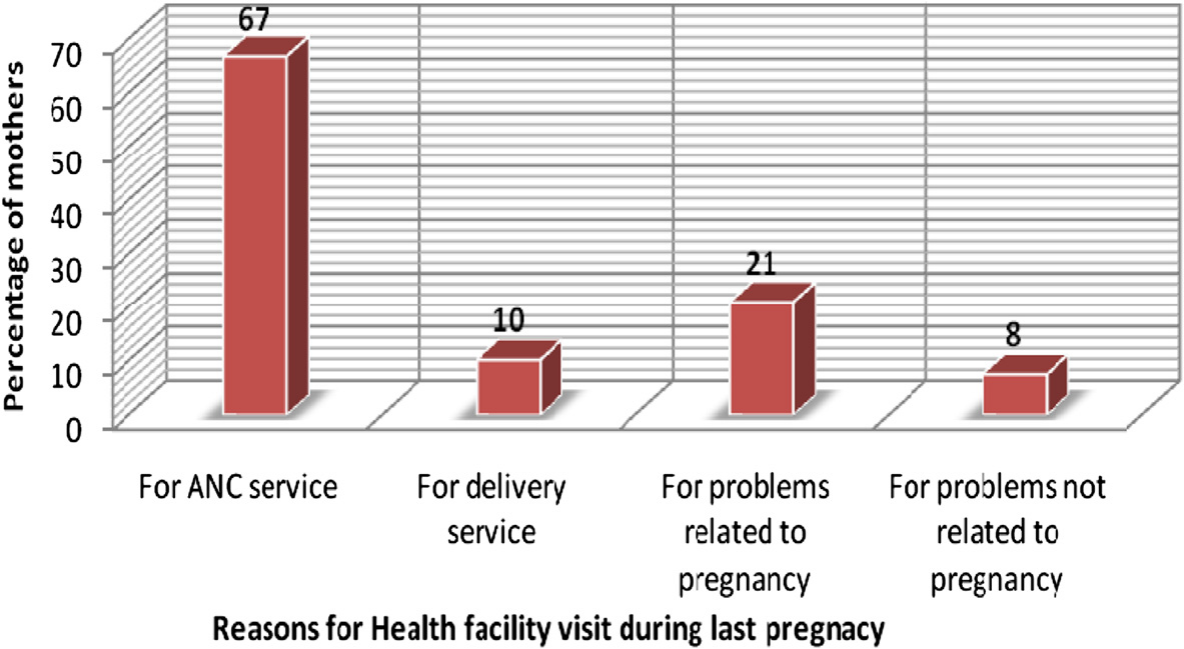
Reasons for health facility visit during the last pregnancy among mothers, Sekela district, North West Ethiopia, Aug. 2010.

Among those mothers who visited health facilities, the reasons for visiting health facilities during the last pregnancy included ANC services, delivery, problems related to pregnancy and problems not related to pregnancy [Figure 2].

**Figure 3:**
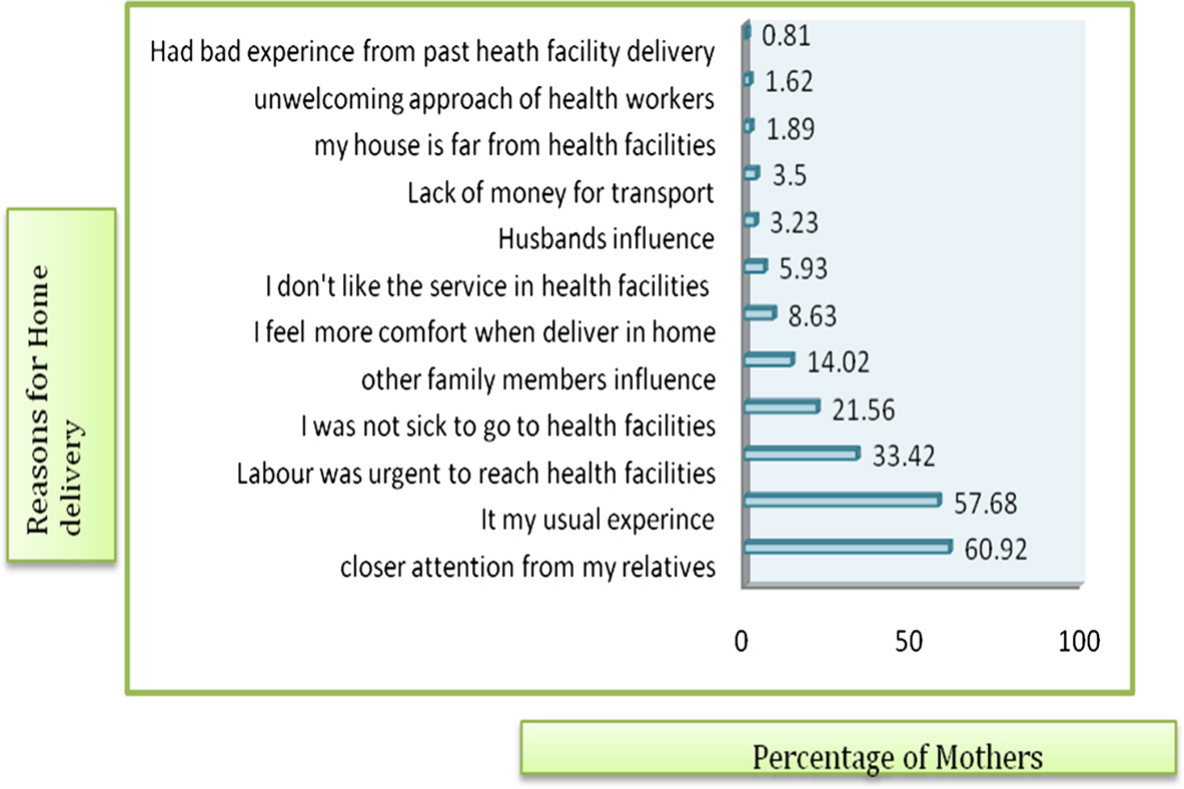
Reasons for home delivery among mothers in Sekela district, North West Ethiopia, Aug. 2010.

Many different reasons were forwarded for home delivery. Two hundred twenty six (60.9%) of the mothers said having closer attention from family members, 214 (57.7%) said delivering at home is my usual experience, 124(33.4%) said labour was short/urgent, 80 (21.6%) said they did not have any problem to go to health facilities, and 52 (14.0%) said influence from family members and others [Figure 3]. Two hundred ten (56.6%) of the mothers scored more than the mean score on knowledge questions which were asked to assess mothers’ knowledge regarding ANC and delivery services at the time of the study. Similarly, 245 (66.0%) of the mothers scored more than the mean score on attitude questions regarding ANC and delivery services while the rest (126, 34.0%) scored less than the mean score [Table 3].

**Table 3:** Knowledge and attitude of the respondents (n = 371) among mothers in Sekela district, North West Ethiopia, August, 2010

**Variables**	**N (%)**
**Knowledge of the Mother on ANC and delivery services**	
Knowledgeable	210(56.6)
Not Knowledgeable	161(43.4)
**Attitude of the Mother on ANC and delivery services**	
Favorable	245(66.0)
Unfavorable	126(34.0)

Regarding preference of the mothers about delivery place during their last pregnancy, 288 (77.6%) preferred to deliver at home, 70 (18.9%) preferred to give birth in health facilities with the assistance of skilled professionals, and 3.5% of the mothers preferred to deliver in their mother’s home. Similarly, 80.6% of their husbands preferred their wife to deliver at their own home, and the rest (19.4%) preferred their wife to deliver in health facilities.

As to the preference of the mothers about delivery attendant, 275 (73.1%) preferred to be attended by family members and 20.7% preferred to be attended by skilled birth attendants during their last pregnancies and 278 (78.3%) of their husbands preferred their wives to be assisted by family members while 18.9% of them preferred them to be assisted by skilled attendants. In line with this, majority (87.1%) of the family members and 88.7% of the community preferred mothers to deliver at home during their last pregnancy [Table 4].

**Table 4:** Preference of the respondents, their husband, family members and the community about place and attendant of delivery during their last pregnancy (n = 371) in Sekela district, North West Ethiopia, August, 2010

**Variables**		**N (%)**
**Preference of the mother about delivery place**	Health facility	70(18.9)
	My home	288(77.6)
	My mother’s home	13(3.5)
Preference of the mother about attendant of delivery	Family members	275(74.1)
	**SBA**	77(20.7)
	**My mother**	19(5.2)
Preference of the husband about place of delivery	**Health facility**	69(19.4)
	**Home**	286(80.6)
Preference of the husband about attendant of delivery	**Family members**	278(78.3)
	**SBA**	67(18.9)
	Others*	10(2.8)
Preference of other family members about place of delivery	**Home**	323(87.1)
	**Health facility**	48(12.9)
Preference of the community about place of delivery	**Home**	329(88.7)
	**Health facility**	42(11.3)
Final decision maker about place of delivery	**Myself**	102(27.5)
	**My husband**	48(5.7)
	**Both of us**	234(63.1)
	Others**	14(3.8)

* *TBA, husband himself.* ** *My mother, family members SBA - skilled birth attendant.*

### Factors associated with institutional delivery service utilization

On bivariate analysis, age of the mother, occupational status of the mother, educational status of the mother, distance from the nearby health center, residence, media of communication, monthly income, number of ANC visits at last pregnancy, gravidity, parity, knowledge and attitude of the mother, ANC visit during last pregnancy, husband’s educational status, husband’s occupational status and obtaining information about delivery place during ANC visit were the factors found to be significantly associated with institutional delivery service utilization [Table 5].

**Table 5:** Bivariate analysis of factors associated with skilled delivery service utilization among mothers in Sekela district, North West Ethiopia, August- 2010

**Variables**	**Delivery service utilization**		**COR (95%CI)**	**P-value**
	**Yes**	**No**		
**Possessing Radio and TV**				
Yes	29	102	3.98(1.01, 7.65)	0.000
No	16	224	1.00	
**Income in month**				
≤408birr	8	89	1.00	0.027
409-693birr	11	79	1.55(0.59, 4.04)	
694-987birr	7	86	0.91(0.31, 2.6)	
>988birr	19	72	2.94(1.21, 7.1)	
**Occupation of the mothers**				
House wife	27	296	1.00	0.000
Gov’t employed	9	5	17.9(5.6,57.4)	
Others **	9	52	1.7(0.77,3.8)	
**Distance**				
<1 hour	42	226	6.2(1.87,20.5)	0.003
≥1hour	3	100	1.00	
**Gravidity**				
1	17	52	3.0(1.3,6.9)	0.003
2-4	17	172	0.9(0.41, 2.0)	
≥5	11	102	1.00	
**Parity**				
1	16	53	3.3(1.7,7.8)	0.01
2-4	20	176	1.2(0.5, 2.8)	
≥5	9	97	1.00	
**Number of ANC visit at last pregnancy**				
One	2	40	1.00	0.047
Two to three	32	133	4.8(1.1,2.9)	
Four and above	11	30	7.3(1.5,3.6)	
**Get information where to deliver during ANC**				
Yes	42	162	3.54(1.0,12.0)	0.042
No	3	41	1.00	
**Attitude of the mothers on ANC and delivery services**				
Favorable	41	204	6.1(2.1,17.5)	0.045
Unfavorable	4	122	1.00	
**Education of the husband**				
Primary and below	27	305	1.00	0.000
Secondary and above	15	8	21.2(8.2,54.4)	
**Occupation of the husband**				
Farmer	13	250	1.00	0.000
Merchant/carpenter	17	54	6.1(2.7,13.7)	
Gov’t and private employed	12	9	25.6(9.2,71.7)	

** Tea & tella seller, daily labourer, merchant, student.

In the multivariable logistic regression analysis only residence of the mother, age at interview, educational status of the mother, ANC visit during last pregnancy and knowledge of the mother were found to be significantly associated with the institutional delivery service utilization [Table 6].

**Table 6:** Multivariate analysis of factors associated with skilled delivery service utilization among mothers in Sekela district, North West Ethiopia, August- 2010

**Variables**	**Delivery service utilization**		**COR (95%CI)**	**AOR (95%CI)**	**P-value adjusted**
	**Yes**	**No**			
**Place of residence**					
Rural	16	280	1.00	1.00	0.001
Urban	29	46	11(5.56,21.89)	4.6(1.91,10.9)	
**Age of the mother**					
15-24	32	78	5.74(1.9,17.16)	4.4(1.15,16.8)	0.000
25-34	9	192	0.66(0.195,2.21)	0.59(0.14,2.6)	
35 and above	4	56	1.00	1.00	
**Educational status of mother**					
Primary education & below	27	321	1.00	1.00	0.000
Secondary and above	18	5	42.8(14.7,124)	11.98(3.46, 41.4)	
**ANC visit during last pregnancy**					
Yes	42	3	8.15(2.47,26.8)	4.26(1.1,16.4)	0.036
No	206	120	1.00	1.00	
**Knowledge of mother on ANC and delivery services**					
Not Knowledgeable	5	156	1.00	1.00	0.044
Knowledgeable	40	170	7.34(2.86,19.1)	2.97(1.1,8.6)	

*Reference category.

-Back ward stepwise multiple logistic regression was used to assess the independent effect of explanatory variables.

Mothers who were urban residents were about 5 times (AOR = 4.6, 95% CI = [1.91, 10.9]) more likely to give birth in health facilities than rural mothers. Mothers with age group of 15–24 years were 4 times more likely to deliver in health institutions than mothers with age group 35 and above (AOR = 4.4, 95% CI = [1.15,16.8]). Mothers with educational level of secondary and above were about 12 times more likely to give birth in health facilities than those with primary education and below (AOR = 11.98, 95% CI = [3.46, 41.4]). ANC visit during last pregnancy was also found to be a strong predictor of institutional delivery service utilization. Mothers who had ANC visit during pregnancy were 4 times more likely to deliver in health facilities than those who did not have ANC visit during last pregnancy (AOR = 4.26, 95% CI = [1.1, 16.4]). Mothers who were knowledgeable on ANC and delivery services were about 3 times more likely to deliver in health institutions than mothers who were not knowledgeable (AOR = 2.97, 95% CI = [1.1, 8.6]).

## Discussion

This community – based study attempted to identify the magnitude of skilled delivery service utilization and associated factors among mothers who gave birth in the last 12 months prior to the study in Sekela District. The study results showed that institutional delivery service utilization was 12.1% in the District and the majority of mothers (87.9%) gave birth at home. The study also revealed that overall delivery assisted by skilled birth attendants was 12.7%. This study finding was higher than National and Amhara region EDHS result of 2005 which was 6% and 3.5% respectively [11]; this might be due to the time gap, i.e., since 2005 there could be improvement in accessing and utilizing the service.

This was also in agreement with the findings of community based cross sectional study among mothers of reproductive age group done in North Gondar Zone in 2002, which was 13.5% [17]. But the finding was much lower than other studies done in Afar region, Asayta and Dupti Towns in which delivery service utilization rate was 54.2% [18]. This large difference could be due study done in Afar Region included only Urban Kebeles in which the negative influence of husbands and family members could be lower than Rural Kebels, and Urban mothers might be able to decide on their own health.

This finding was also lower than cross sectional studies done in Zambia and Tanzania among mothers who had delivered babies within one year prior to the survey which showed that the rate of utilization of a health facility for childbirth was 42.8% and 46.7%, respectively [19,20]. This difference might be attributable to the economic difference that mothers in these countries could have better socio economics and educational status. In addition, in these countries there might be better ANC and family planning services utilization. Moreover, there could be differences in culture and the studies were conducted among urban populations, this could positively influence delivery service utilization in these countries.

The current study also revealed that mothers’ place of residence was strongly associated with institutional delivery service utilization. Mothers who lived in Urban Kebeles were five times more likely to deliver in health facilities than those who live in Rural Kebeles. This finding was consistent with the study done in Ethiopia by Yared M. and Asnaketch M. using EDHS 2000 data which revealed that mothers living in Addis Ababa and other urban areas of the country were 40 and 9 times more likely to deliver in health institutions than rural dwellers, respectively [21]. The reason for these corroborative findings might be due the fact that in urban areas the proportion of mothers with education is higher, accessibility of the services with minimal distance and transport, and mothers could have better decision making autonomy, good knowledge of pregnancy and delivery complications, and better access to information than rural mothers.

Mothers with age range 15–24 years were about 4 times more likely to give birth in health institutions than mothers with age 35 years and above. This finding was in agreement with the study done in North Gondar Zone that showed utilization of delivery service was higher in younger age group (15–24 years) as compared to lower utilization of the services by mothers with age groups 25–34 years, and 35 years and above [17]. This finding was also consistent with the study done using EDHS data by Yared M. and Asnakech M. and the study in Asayta and Dupti which showed younger mothers were more likely to utilize the service than older ones [18,21]. The possible explanations might be younger women are more likely to be literate than older women and older women consider that giving birth at home is not risky as they have previously experienced birth there.

Educational status of the mother had also significant association with institutional delivery service utilization. Mothers who attended secondary education and above were 12 times more likely to utilize delivery service than those mothers who had primary education and below. This finding was similar to other studies by Yared and Asnaket using EDHS data, and studies done in Bangladesh in 2007, Zambia and North Gondar Zone which showed mothers with higher educational status were more likely to utilize delivery care service. Supporting finding of the study conducted in North Gondar indicated that mothers with educational status of secondary and above were 2.3 times more likely to delivery in health facilities than mothers with lower educational level [17,19,21,22].

This study also revealed that mothers who visited ANC during last pregnancy were about four times more likely to deliver in health facilities than mothers who did not visit ANC. Corroborative studies done in North Gondar Zone, Nepal, and India revealed that those mothers who attended ANC were more likely to utilize delivery care services than those who did not attend. The study conducted in North Gondar showed that women who did not visit health facilities were 0.5 less likely to deliver in health facilities than those who visited health facilities [17,23,24].

Knowledge of the mothers about pregnancy and delivery related the services, advantages of these services, pregnancy and delivery related complications and mothers susceptible to these complications was found to be significantly associated with delivery service utilization. Mothers who had good knowledge were about three times more likely to deliver in health institutions than mothers who had poor knowledge. This finding was similar to the study done in Tanzania that showed knowledge of mothers on pregnancy risk factors had significant association with use of skilled care at delivery even after controlling for confounding factors [20].

## Conclusions

Very low (12.1%) institutional delivery service utilization was observed in the study area though 66.8% of the mothers attended ANC services during their last pregnancy. A large proportion (87.9%) of mothers gave birth at home without a skilled attendant. The majority (80.0%) of mothers who gave birth at home were assisted by family members and relatives. Closer attention and care from family members and relatives, delivering at home is usual experience, having much freedom at home during delivery, influence from the husband and other family members, disliking the services provided at the health facilities, lack of sufficient knowledge about the services, labour was unexpected/short and absence of problem were the main reasons given by mothers not attending health care delivery. Factors such as being urban resident, age at interview, ANC visit during the last pregnancy, educational status of the mother and knowledge of the mothers on pregnancy and delivery services were significantly associated with skilled delivery service utilization.

### Recommendations

Health improvement strategies with a focus on increasing mothers’ knowledge and awareness about the services given, the benefits of these services, and complications related to pregnancy and delivery, should be designed and implemented. Skilled attendants at Health Post level should be trained and assigned to provide home delivery. As a short term solution, training all Health Extension Workers so that they could have some midwifery skills is highly recommended. Information, education, communication, and empowering mothers is essential, and could help them in decision making regarding their own health, being committed to use the services and able to persuade their partner and family members if they encountered opposition. Information on the complications of pregnancy and delivery and on the importance of using either the institutional delivery service, or skilled midwifery assistance in the home, at every childbirth should be given to every mother who came to health facility in general and at ANC visits in particular.”

### Strength and weakness of the study

The main strength of this study is that, being community based, it could reflect the actual experience of the mothers during the study period. However, the cross sectional nature of the study means that it cannot establish causal associations.

## Endnotes

^a^Health post – the smallest unit of health facility at the lower level of administration (kebele).

^b^Kebele is the smallest administrative unit of the Federal Democratic Republic of Ethiopia.

## Abbreviations

ANC, Antenatal Care; APH, Ante Partum Hemorrhage; EDHS, Ethiopian Demographic and Health Survey; ETB, Ethiopian Birr; FMOH, Federal Ministry of Health; HEWs, Health Extension Workers; MDG, Millennium Development Goals; MMR, Maternal Mortality Ratio; PPH, Post Partum Hemorrhage; SBAs, Skill Birth Attendants; SD, Standard Deviation; SSA, Sub Saharan Africa; TBAs, Traditional Birth Attendants; TTBAs, Trained Traditional Birth Attendants; WHO, World Health Organization.

## Competing interests

The authors have declared that they have no competing of interests.

## Authors’ contributions

AS designed the study, participated in the data collection, performed analysis and interpretation of data and drafted the paper and prepared the manuscript.FM and SMW assisted in the design, approved the proposal with some revisions, participated in data analysis and revised subsequent drafts of the paper. All authors read and approved the final manuscript.

## Pre-publication history

The pre-publication history for this paper can be accessed here:

http://www.biomedcentral.com/1471-2393/12/74/prepub
